# Bacterial Enteric Infections Detected by Culture-Independent Diagnostic Tests — FoodNet, United States, 2012–2014

**Published:** 2015-03-13

**Authors:** Martha Iwamoto, Jennifer Y. Huang, Alicia B. Cronquist, Carlota Medus, Sharon Hurd, Shelley Zansky, John Dunn, Amy M. Woron, Nadine Oosmanally, Patricia M. Griffin, John Besser, Olga L. Henao

**Affiliations:** 1 Division of Foodborne, Waterborne, and Environmental Diseases, National Center for Emerging and Zoonotic Infectious Diseases, CDC; 2 Colorado Department of Public Health and Environment; 3 Minnesota Department of Health; 4 Connecticut Department of Public Health; 5 New York State Department of Health; 6 Tennessee Department of Health; 7 Georgia Department of Public Health

The increased availability and rapid adoption of culture-independent diagnostic tests (CIDTs) is moving clinical detection of bacterial enteric infections away from culture-based methods. These new tests do not yield isolates that are currently needed for further tests to distinguish among strains or subtypes of *Salmonella*, *Campylobacter*, Shiga toxin–producing *Escherichia coli*, and other organisms. Public health surveillance relies on this detailed characterization of isolates to monitor trends and rapidly detect outbreaks; consequently, the increased use of CIDTs makes prevention and control of these infections more difficult (1–3). During 2012–2013, the Foodborne Diseases Active Surveillance Network (FoodNet*) identified a total of 38,666 culture-confirmed cases and positive CIDT reports of *Campylobacter, Salmonella, Shigella*, Shiga toxin–producing *E. coli*, *Vibrio*, and *Yersinia*. Among the 5,614 positive CIDT reports, 2,595 (46%) were not confirmed by culture. In addition, a 2014 survey of clinical laboratories serving the FoodNet surveillance area indicated that use of CIDTs by the laboratories varied by pathogen; only CIDT methods were used most often for detection of *Campylobacter* (10%) and STEC (19%). Maintaining surveillance of bacterial enteric infections in this period of transition will require enhanced surveillance methods and strategies for obtaining bacterial isolates.

Culturing of organisms has been the mainstay of clinical diagnostic testing for bacterial enteric pathogens. Currently, isolates obtained from culture are forwarded from clinical laboratories to public health laboratories, where additional testing is performed, including antimicrobial susceptibility testing, serotyping, pulsed-field gel electrophoresis, and whole genome sequencing. Advances in clinical microbiology have led to the emergence of culture-independent diagnostic tests, such as those that detect the presence of a specific antigen or the DNA of an organism. Many of these new tests will likely improve patient care by allowing rapid diagnosis, improving sensitivity and simplicity, lowering costs, and by detection of a wider range of pathogens. However, current culture-independent diagnostic methods do not have subtyping ability that enables determination of antimicrobial resistance, detection of clusters of illness, and monitoring of trends. Currently, the extent of culture-independent diagnostic practices by clinical laboratories and the future impact on public health surveillance are unknown.

To address these knowledge gaps, in 2010 FoodNet began to survey clinical laboratories serving surveillance catchment area residents on the use of new testing methods to detect enteric pathogens in stool specimens. In 2011, FoodNet expanded surveillance to include the collection of epidemiologic and pertinent laboratory data on both culture-confirmed and positive CIDT reports of *Campylobacter*, *Salmonella*, Shiga toxin–producing *Escherichia coli* (STEC), *Shigella*, *Vibrio*, and *Yersinia* infections. Two data sources were examined: a survey of clinical laboratories conducted during January–March 2014 and surveillance data during January 2012–December 2013. Culture-confirmed infections were defined as the isolation of a bacterial enteric pathogen from a clinical culture from a patient residing in the surveillance area. A positive CIDT report was defined as the detection of the enteric pathogen, or for STEC, Shiga toxin or the genes that encode a Shiga toxin, in a stool specimen or enrichment broth using a CIDT. In some instances, stool culture was performed in conjunction with CIDT. All reports were classified into four mutually exclusive categories, based on whether stool culture was performed and culture results: culture-positive only, CIDT-positive and culture-positive, CIDT-positive and culture-negative, and CIDT-positive and no culture. CIDTs were categorized into four test types: commercial antigen-based tests (Food and Drug Administration [FDA]–approved), commercial DNA-based syndrome panels (FDA-approved), laboratory-developed tests (LDTs†) typically used in a single clinical laboratory, and LDTs used at a public health laboratory.§ Incidence was calculated using U.S. Census estimates of the surveillance area populations for 2012 and 2013. Because there were few differences between 2012 and 2013 data, this report combines surveillance data for both years.

## Survey of FoodNet Clinical Laboratories, 2014

The use of CIDTs by clinical laboratories varied by pathogen; CIDT methods were used most often for detection of *Campylobacter* and STEC. During January–March 2014, 446 (67%) of 664 of clinical laboratories serving the FoodNet surveillance area tested stool specimens for *Campylobacter*. Of these laboratories, 379 (85%) used only culture methods to detect *Campylobacter*, 45 (10%) used only CIDTs, and 22 (5%) used both culture and CIDTs. Among laboratories using CIDTs to detect *Campylobacter*, 62 (90%) used commercial antigen-based methods, three used commercial DNA-based syndrome panels, and two used LDTs.¶ Of the 395 (60%) clinical laboratories that tested stool specimens for STEC, 187 (47%) used both culture and CIDTs, 135 (34%) used only culture, and 73 (19%) used only CIDTs. Among laboratories using CIDTs to detect Shiga toxin or the genes that encode the toxins, 258 (99%) used commercial antigen-based tests, three used commercial DNA-based syndrome panels, and two used LDTs.** Of the 453 (68%) laboratories that tested clinical specimens for *Salmonella*, six (1.3%) used CIDTs; among these, three used commercial DNA-based syndrome panels, and three used LDTs.††

## FoodNet Surveillance, 2012–2013

**FIGURE 1:**
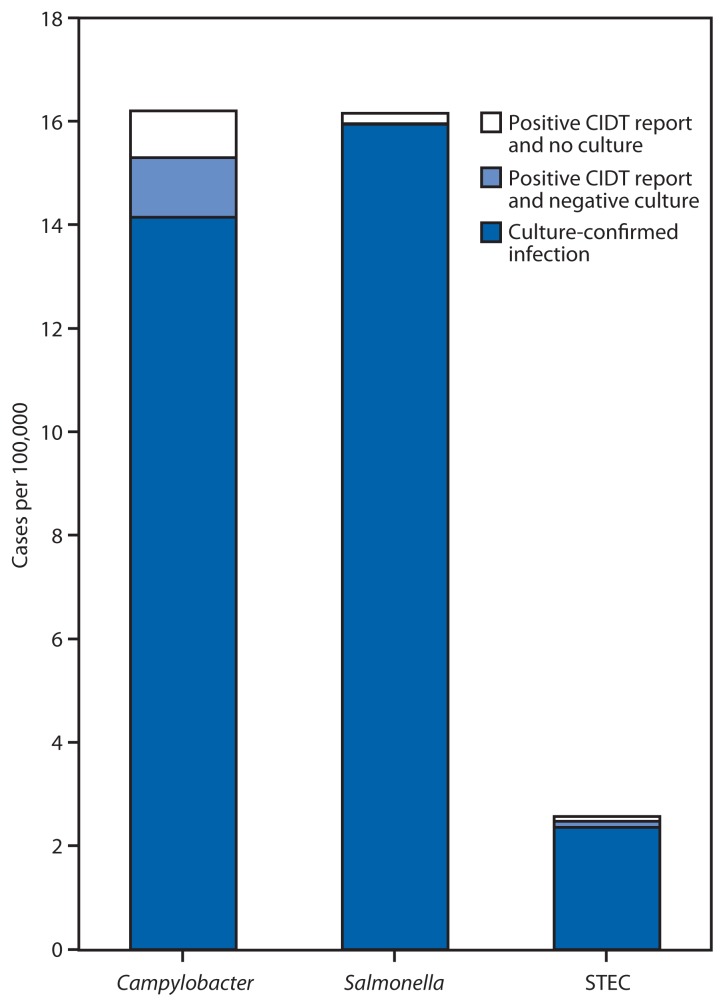
Incidence of culture-confirmed bacterial infections and positive CIDT reports, by selected pathogen — FoodNet, United States, 2012–2013 **Abbreviations:** CIDT = culture- independent diagnostic test; STEC: Shiga toxin–producing *Escherichia coli*.

FoodNet identified 38,666 culture-confirmed cases and reports of positive CIDTs during 2012–2013 (Table). Among the 5,614 reports of positive CIDTs, 2,595 (46%) were not confirmed by culture, either because a culture did not yield the pathogen or because the specimen was not cultured. Among the 2,497 positive CIDT reports of *Campylobacter*, 539 (22%) were confirmed by culture, 1,099 (44%) were culture-negative, and 859 (34%) had no culture. Among the 2,409 positive CIDT reports of STEC,§§ 2,205 (92%) were confirmed by culture, 110 (5%) were culture-negative, and 94 (4%) had no culture. The Shiga toxin–positive result was confirmed for 2,241 (90%) of 2,494 enrichment broths sent to a public health laboratory. Among 308 positive CIDT reports of *Salmonella*, 115 (37%) were confirmed by culture, eight (3%) were culture-negative, and 185 (60%) had no culture. The incidence of culture-confirmed infections with *Campylobacter* was 14.1 per 100,000 population, compared with 2.1 for positive CIDT reports with no culture or negative culture. For *Salmonella*, the incidence was 16.0 per 100,000 population for culture-confirmed infections and 0.2 for positive CIDT reports with no culture or negative culture, and for STEC, the incidence was 2.4 per 100,000 population for culture-confirmed infections and 0.21 for positive CIDT reports with no culture or negative culture (Figure 1).

**FIGURE 2:**
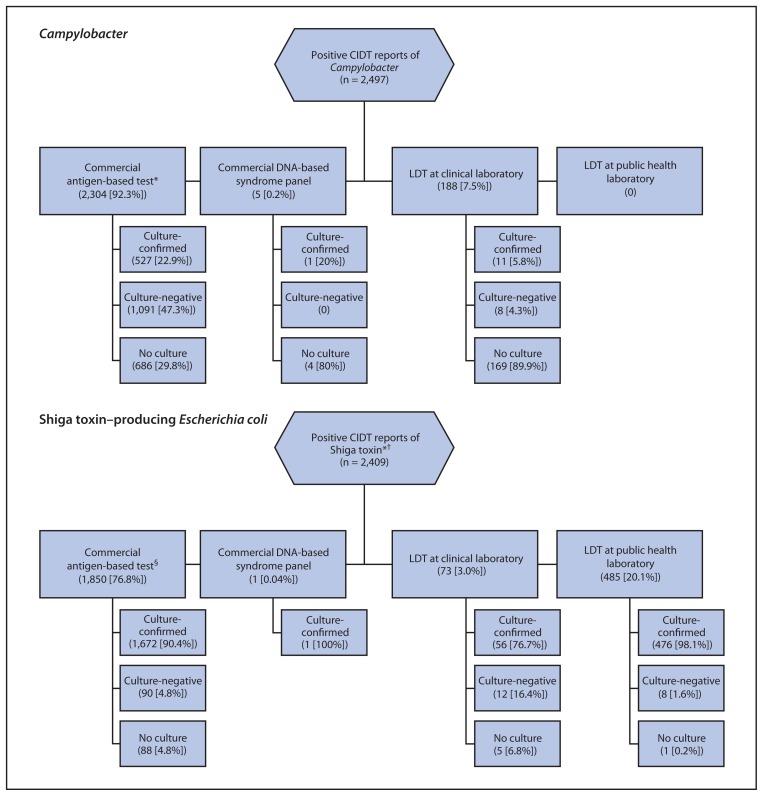
Positive CIDT reports of *Campylobacter* and Shiga toxin–producing *Escherichia coli*, by test type and culture result — FoodNet, United States, 2012–2013 **Abbreviations:** CIDT = culture- independent diagnostic test; LDT = laboratory-developed test. * Excludes 274 Shiga toxin–positive reports from clinical laboratories that were Shiga toxin–negative at a public health laboratory and 53 reports of detection of O157 antigen without a test result for Shiga toxin. ^†^ For instances in which a positive result from a single specimen was reported from more than one laboratory (e.g., clinical laboratory and public health laboratory), test type was categorized according to the test type used for initial detection. ^§^ Conducted at a clinical laboratory or public health laboratory.

Among 2,497 positive CIDT reports of *Campylobacter*, 2,304 (92.3%) were detected using commercial antigen-based tests; among 1,618 antigen-positive specimens that were cultured, 1,091 (67%) were culture-negative. Among 2,409 positive CIDT reports of STEC, 1,850 (77%) were detected using commercial antigen-based tests (Figure 2). Among 308 positive CIDT reports for *Salmonella*, 303 (98%) were detected using an LDT in a clinical laboratory.

## Discussion

FoodNet surveillance indicates CIDTs are being used in clinical care, currently most often to detect *Campylobacter* and STEC infections. Overall, a concerning proportion of positive CIDT reports were not confirmed by culture, either because the specimen was not cultured or because a culture did not yield the pathogen. The use of CIDTs for specific pathogens has increased over time. The use of the newer generation commercial DNA-based syndrome panels has been modest to date. However, with many recent approvals of CIDTs that offer advantages to clinicians and clinical laboratories over traditional culture-based methods, many clinical laboratories are in the process of switching to CIDTs and accelerated use is anticipated over the next year (FoodNet, unpublished data, 2014). Taken together, these findings warrant increased attention to surveillance for all bacterial enteric pathogens and critical examination of the results of CIDTs.

*Campylobacter* has been the most common pathogen detected using a CIDT; however, this could change as more laboratories adopt commercial DNA-based syndrome panels. Laboratory practices to detect *Campylobacter* infections have changed at FoodNet sites; the proportion of clinical laboratories using a CIDT increased from fewer than 3% of clinical laboratories in 2004 to 15% in 2014 (4). The corollary to this use by laboratories is that positive CIDT reports accounted for more than 16% of all *Campylobacter* reported (culture-confirmed infections and positive CIDT reports without culture confirmation). Among positive CIDT reports, almost all were results from commercial antigen-based tests, and almost half of the associated specimens were culture-negative. The high proportion of culture-negative reports might be explained by poor transport stability of the organism, but there is evidence the reports might represent false-positive results because of the widespread use of antigen-based tests with poor test performance (5,6). The impact of the variability in CIDT characteristics (i.e., sensitivity and specificity) on clinical practice is unknown.

CIDTs are becoming more widely used for the diagnosis of STEC infections. Comparing clinical laboratory practices in 2007 and 2014, the use of antigen-based and DNA-based methods to detect Shiga toxin or the genes encoding the toxins increased from 11% to 60% of clinical laboratories (7). A positive CIDT report was associated with almost all STEC reports, and most of these reports (90.4%) were confirmed by culture. There are established best-practice recommendations, and in most FoodNet sites,¶¶ state requirements for the referral of Shiga toxin–positive broths to public health laboratories for confirmation (8,9). The high proportion of CIDTs performed in conjunction with culture and confirmed at a public health laboratory demonstrates that laboratory guidance and submission requirements are effective strategies to promote the testing of specimens by culture and the flow of isolates or clinical specimens to public health laboratories.

Quantifying the impact of CIDTs on trends in disease incidence and burden is complicated because of important limitations of the understanding of CIDTs and possible changes in laboratory practices surrounding them. First, it is difficult to draw conclusions from increases or decreases in the number of reports partly because many types of CIDTs are being used. Test performance characteristics differ among CIDTs and might differ among patient populations. Second, trends would be affected if CIDT testing practices were different from culture; for example, if CIDTs were used more frequently for specific patient populations or for different clinical indications. Finally, available CIDTs for enteric pathogens do not have subtyping capacity.

As more clinical laboratories adopt CIDTs, the collection and detailed characterization of bacterial isolates that support public health activities will fall more heavily on public health laboratories. The increased reliance on CIDTs will create a burden for public health laboratories and will have a significant impact on clinical practice, outbreak detection, and the ability to monitor disease burden and trends. Public health surveillance programs rely on the ability to distinguish among strains and serotypes of pathogens to detect foodborne outbreaks and monitor the effectiveness of specific public health and food safety interventions by regulatory agencies and the food industry. To maintain public health surveillance of foodborne and other bacterial enteric diseases and to maintain the quality of clinical decision-making, it will be necessary to 1) enhance surveillance methods to gather sufficient information on CIDT reports (e.g., type and brand of test) to allow critical examination of the data to assess case definitions and to inform both evidence-based best clinical and laboratory practices, 2) encourage and implement reflex culturing (culturing of a specimen with a positive CIDT result) at clinical laboratories or submission of appropriate specimens for culture to public health laboratories, and 3) develop further strain characterization methods that are themselves culture-independent for improved clinical management and public health surveillance.

**What is already known on this topic?**

Culture-independent diagnostic tests (CIDTs) are increasingly used by clinical laboratories to diagnose bacterial enteric infections. CIDTs do not yield isolates, which are needed for further characterization by current methods, including antimicrobial susceptibility testing, serotyping, pulsed-field gel electrophoresis, and whole genome sequencing.

**What is added by this report?**

FoodNet surveillance indicates CIDTs are being used in clinical care, currently most often to detect *Campylobacter* and STEC infections. During 2012–2013, the Foodborne Diseases Active Surveillance Network (FoodNet) identified a total of 38,666 culture-confirmed cases and positive CIDT reports of *Campylobacter, Salmonella, Shigella*, Shiga toxin–producing *E. coli*, *Vibrio*, and *Yersinia*; among the 5,614 positive CIDT reports, 2,595 (46%) were not confirmed by culture, either because the specimen was not cultured or because a culture did not yield the pathogen. In addition, a 2014 survey of clinical laboratories serving the FoodNet surveillance area indicated that use of CIDTs by the laboratories varied by pathogen; only CIDT methods were used most often for detection of *Campylobacter* (10%) and STEC (19%).

**What are the implications for public health practice?**

Although CIDTs provide many advantages over culture to improve patient care, the increased reliance on CIDTs, coupled with the public health need to obtain subtype information about isolates to detect outbreaks and monitor disease trends, likely will result in a burden on public health laboratories.

## Figures and Tables

**TABLE:** Number of culture-confirmed cases and positive culture-independent diagnostic test (CIDT) reports (N = 38,666), by selected pathogens and culture results — FoodNet, United States, 2012–2013

Pathogen	Culture-positive only		Positive CIDT reports						Total culture-confirmed infections and positive CIDT reports

			CIDT-positive and culture-positive		CIDT-positive and culture-negative		CIDT-positive and no culture		
				
	No.	(%)	No.	(%)	No.	(%)	No.	(%)	No.
*Campylobacter*	12,894	(83.8)	539	(3.5)	1,099	(7.1)	859	(5.6)	15,391
*Salmonella*	15,034	(98.0)	115	(0.7)	8	(0.1)	185	(1.2)	15,342
*Shigella*	4,312	(91.8)	160	(3.4)	27	(0.6)	197	(4.2)	4,696
STEC*†	34	(1.4)	2,205	(90.3)	110	(4.5)	94	(3.8)	2,443
*Vibrio*	446	(98.0)	0	—	5	(1.1)	4	(0.9)	455
*Yersinia*	332	(98.0)	0	—	2	(0.6)	5	(1.4)	339
**Total**	**33,052**	**(85.5)**	**3,019**	**(7.8)**	**1,251**	**(3.2)**	**1,344**	**(3.5)**	**38,666**

**Abbreviation:** STEC = Shiga-toxin–producing *Escherichia coli.*

* Excludes 274 Shiga toxin–positive reports from clinical laboratories that were Shiga toxin–negative at a public health laboratory.

† Excludes 53 positive reports of detection of O157 antigen without testing for Shiga toxin.

## Footnotes

* FoodNet is a collaborative program among CDC, 10 state health departments, the Food Safety and Inspection Service and the Food and Drug Administration. Since 1996, FoodNet’s core work has been active, population-based surveillance for culture-confirmed infections caused by *Campylobacter, Listeria, Salmonella*, Shiga toxin-producing *Escherichia coli* (STEC), *Shigella, Vibrio*, and *Yersinia* and laboratory-confirmed infections of *Cryptosporidium* and *Cyclospora*. The 10-site FoodNet surveillance area includes approximately 15% of the U.S. population (estimated 48 million in 2013). FoodNet site personnel regularly contact clinical laboratories to ascertain all laboratory-confirmed infections in residents of the surveillance area.

† FDA defines an LDT as an in vitro diagnostic test that is manufactured by and used within a single laboratory. LDTs do not require FDA approval, and there are no premarket review and quality system requirements on LDTs currently. However, FDA has released draft guidance for regulatory oversight of LDTs for public comment. Available at http://www.fda.gov/downloads/MedicalDevices/DeviceRegulationandGuidance/GuidanceDocuments/UCM416685.pdf.

§ All reported LDTs used at public health laboratories were DNA-based assays to detect Shiga toxin 1 and Shiga toxin 2 genes for diagnosis and detection of STEC infection.

¶ The reported commercial antigen-based tests used to detect Campylobacter included ImmunoCard STAT! CAMPY (Meridian), ProSpecTCampylobacter Microplate (Remel), and Premier CAMPY (Meridian), and the commercial DNA-based syndrome was xTAG GPP (Luminex).

** The reported commercial antigen-based antigen tests used to detect STEC included ImmunoCard STAT! EHEC (Meridian), Premier EHEC (Meridian), Shiga Toxin Quik Chek (Alere), ImmunoCard STAT! *E.coli* O157 Plus (Meridian), and ProSpecT Shiga Toxin *E. coli* (Remel), and the commercial DNA-based syndrome panels were xTAG GPP (Luminex). Three laboratories used both commercial antigen-based tests and commercial DNA-based syndrome panels to detect STEC.

†† The reported commercial DNA-based syndrome panel used to detect *Salmonella* was xTAG GPP (Luminex). There are no commercial antigen-based tests to detect *Salmonella* in stool specimens.

§§ The number of positive CIDT reports of STEC excludes 274 Shiga toxin–positive reports from clinical laboratories that were Shiga toxin–negative at a public health laboratory and 53 reports of detection of O157 antigen without a test result for Shiga toxin.

¶¶ California, Connecticut, Maryland, Minnesota, New Mexico, New York, Oregon, and Tennessee have state laws or regulations for the submission of Shiga toxin–positive broths to the state public health laboratory.
